# Impact of Neuro Physiotherapeutic Reformation in a Teenager Agonizing With Guillain-Barre Syndrome Linked With COVID-19 Infection

**DOI:** 10.7759/cureus.28650

**Published:** 2022-08-31

**Authors:** Pallavi Harjpal, Suchitra Menon, Rakesh K Kovela, Moh'd Irshad Qureshi

**Affiliations:** 1 Neuro Physiotherapy, Ravi Nair Physiotherapy College, Wardha, IND; 2 Neuro Physiotherapy, Datta Meghe Institute of Medical Sciences, Wardha, IND; 3 Physiotherapy, NITTE Institute of Physiotherapy, NITTE (Deemed to be University), Mangalore, IND

**Keywords:** case report., rehabilitation, early physiotherapy, covid-19 retro, guillain - barre syndrome

## Abstract

Coronavirus disease 2019 (COVID-19) has spread around the globe. The most common symptoms associated with this are usually respiratory, but different central nervous system manifestations have been reported. There are many cases of Guillain-Barre syndrome (GBS) post-COVID-19. However, only a few simultaneous afflictions of COVID-19 with GBS have been reported. Therefore, our study aims to investigate a case of GBS along with COVID-19 infection in India. A 22-year-old male with no medical history presented with fever along with global weakness and breathing difficulty. There was no history of travel. At the time of admission, he had developed quadriparesis and had muscular strength of 2/5 in bilateral lower limbs and 3/5 in bilateral upper limbs. When the patient developed breathing difficulty, he was transferred to the intensive care unit. The cerebrospinal fluid evaluation showed albumin-cytological dissociation, and a nerve conduction study was done. The patient was managed by neuro physiotherapy 34 days after COVID-19 exposure. After proper physiotherapy and rehabilitation, the patient was able to return to his college life.

## Introduction

The severe acute respiratory syndrome coronavirus 2 (SARS-CoV-2) that causes coronavirus disease 2019 (COVID-19) primarily affects the respiratory system but has also been linked to several neurological symptoms, including headache, confusion, myalgia, dizziness, and loss of taste and smell [1]. Though the first reported case in Wuhan, China showed a para-infectious presentation, this has been characterized as a probable uncommon sequela of COVID-19 [2]. Acute inflammatory demyelinating polyradiculopathy, i.e., Guillain-Barré syndrome (GBS) is defined by symmetrical, increasing limb weakness, areflexia on examination, sensory complaints, and, in some cases, facial paralysis that develops over several days and weeks [3]. GBS is an uncommon, immune-mediated, post-infectious neuropathy that often causes progressive weakening. According to preliminary reports, GBS can be a rare consequence of COVID-19 [4,5]. Since then, the number of cases has been increasing day by day not only in India but also worldwide. There are several high-quality studies suggesting the correlation of GBS with COVID-19, and it is necessary to test for COVID-19 in a patient reporting GBS [6]. Here, we present a unique case of COVID-19 simultaneously with GBS. Because these conditions have overlapping clinical features, such as respiratory involvement and limb weakness, the diagnosis of one may be overlooked by the other. Proper diagnosis and early treatment are required for both.

Cardio-respiratory training and neurorehabilitation, including active-assisted training, and progressing to strengthening, have proved to be effective in previous studies [3]. The Hughes severity score of GBS patients provides a measure of disability [7], and this was used to tailor neurorehabilitation according to the patient’s initial assessment, together with physiotherapy and regular monitoring of the vitals. Within a month the patient was able to become functionally independent without any residual weakness. Early physiotherapy plays an important role in regaining functional independence in such patients.

## Case presentation

Patient information

A 22-year-old male was referred to our hospital from a primary health care center. His presenting symptom was a fever, for which he took paracetamol and antibiotics from the local hospital and got relief. Five days later, he again complained of a fever in the morning, and in the afternoon, he started complaining of sudden weakness in all four limbs, for which he went to a local hospital, where he was assessed and referred to our hospital. The next day he tested positive for COVID-19 (as a protocol before hospital admission) and was placed in isolation for seven days. He was provided with intravenous immunoglobulin but soon developed respiratory distress necessitating transfer to the ICU for oxygen support, which he received for a total of 10 days. He was initially treated with intravenous immunoglobulin and improved. After 15 days, he tested negative and was transferred to the medical ward four days later. The patient was then referred for physiotherapy 34 days after the initial COVID-19 symptom onset.

Clinical findings

The patient was of mesomorphic build, well-oriented, and had intact sensations in bilateral upper and lower extremities. Muscle power was reduced in the right upper and bilateral lower limbs suggestive of weakness with a grade of 4/5 on the Medical Research Council muscle scale in the shoulder and elbow, 3/5 in the wrist, a grade of 3/5 in the hips and knee muscles, and 2/5 over his ankle muscles. Lower limbs were involved more than upper limbs. Deep tendon reflexes were diminished in lower limbs but preserved in bilateral biceps, triceps, and supinators. Abdominal reflexes were absent, bowel and bladder function were affected, and the patient was catheterized. Plantar reflexes were absent on the right and flexor on the left. Breathing was normal with no secretions, but he had difficulty taking deep breaths and was using his accessory muscle (sternocleidomastoid), which was the main reason for early fatigue. Air entry was reduced in bilateral lower lobes. 

Clinical diagnosis

Both the comprehensive metabolic panel and complete blood count were within normal range. The patient's nasopharyngeal swab was sent for SARS-CoV-2 identification on the same day of admission in light of the current global pandemic and his history of fever. One day following hospitalization, a lumbar puncture was performed for cerebrospinal fluid (CSF) analysis. He had albumin-cytological dissociation in his CSF. The patient was identified as having a mild dual diagnosis of COVID-19 and GBS. He was diagnosed with a case of sensory-motor polyneuropathy with nerve conduction velocity findings, which showed that compound muscle action potential amplitude could not be elicited in the bilateral peroneal nerve and was reduced, with prolonged distal motor latency and conduction velocity within the normal limit in the bilateral median, ulnar, and tibial nerves. F-min latency could not be elicited in the bilateral median, ulnar, tibial, and peroneal nerves. Sensory nerve action potential amplitude could not be elicited in the bilateral sural and right ulnar nerve and was reduced in bilateral median nerves

Physiotherapy functional assessment

The functional independence measure score taken on the first day of physiotherapy evaluation was 68/126, and the Hughes severity scale score was 4/6 (confined to bed). By then it was evident that the patient was dependent on caregivers for his activities of daily living (ADL).

The timeline of events in the ICU and wards is shown in Table 1.

**Table 1:** Timeline of events. ICU: intensive care unit, IV: intravenous, T.: tablet, OD: once daily, BD: twice daily, ADL: activities of daily living, IADL: instrumental activities of daily living.

S. No.	Date of Events	Consultation	Findings	Suggestions
1.	On admission (COVID-19 positive)	Emergency	Bilateral lower limb and upper limb weakness, blurring of vision, diplopia, and COVID-19	Inj. methylprednisolone: 1 mg IV OD; Inj. piptaz: 4 mg IV OD; Inj. Emeset: 4 mg IV; T. favipiravir: 1,800 mg for 1 day; T. Limcee: OD; T. Zincovit OD
2.	26/09/2021	Isolation	NCV revealed sensory-motor polyneuropathy	Neuromonitoring, watch for SPO_2_ and respiratory rate. T. favipiravir: 800 mg BD (2^nd^ -7^th^ day) T. Limcee OD T. Zincovit OD IV IG 25 mg (03/10/2020 to 08/10/2020) IV IG 5 mg (09/10/2020)
3.	03/10/2021	Medicine ICU	Difficulty in breathing and maintaining saturation, weakness persisting	On O_2_ via facemask for 7-10 days. methylprednisolone 1 g Inj. meropenem 1 g Inj. levofloxacin 500 mg BD Inj. pantoprazole OD
3.	14/10/2021	Medicine ward	COVID-19 negative, bilateral lower limb, and upper limb weakness	Tab pantoprazole IV NS Ophthalmology call- no diplopia was found. Neuro physiotherapy call
4.	22/10/2021	Neuro physiotherapist	Acute inflammatory demyelinating polyneuropathy -GBS without the involvement of cranial nerves	The physiotherapy session started and continued till discharge i.e., 14/11/2020 with a proper home exercise program
5.	02/12/2021	Neuro physiotherapist	Difficulty performing complex ADL and IADL	Strengthening exercises, gait training, fine motor training

Physiotherapy interventions

After 34 days with symptoms of GBS due to COVID-19, the patient had the effects of de-conditioning due to prolonged bed rest. The chest was clear on assessment, but as the patient had a history of recent COVID-19 infection, chest physiotherapy (breathing exercises, thoracic expansion exercises, pursed-lip breathing along with proper positioning) was provided to increase the chest excursion and reduce the level of stress. For maintaining joint mobility and integrity, range of motion exercises along with a regular change in position were taught to the patient. The treatment protocol is presented in Table 2. This protocol was provided once daily to the patient, with 10 repetitions of each exercise and proper rest periods in between. Along with this treatment protocol, the patient was advised to perform active limb movement, breathing exercises, and stress ball exercises during the evening hours.

**Table 2:** Physiotherapy intervention protocol. ADL: Activities of daily living

Problem identified	Probable cause	Goal Framed	Physiotherapy Intervention
Decreased air entry into the lungs	Weakness of the diaphragm and intercostal muscles	Mr. X will be able to perform the mild strenuous activity without excursion within two weeks	Diaphragmatic breathing (Figure 1C), thoracic expansion exercises, pursed lip breathing, and incentive spirometry
Decreased range of motion	Prolonged bed rest	Mr. X will be able to perform activities in full range without any difficulty within two weeks	Active range of motion exercise involving bilateral upper and lower extremities and calf stretching
Weakness of extremity muscles	Decreased nerve conduction	Mr. X will be able to regain the reduced strength in his limbs within two weeks of intervention	Plan for giving electrical stimulation to increase muscle performance
Inappropriate posture	Bedridden for many days postoperatively	Proper posture will be gained by the patient by the end of two weeks	Chest binders and positioning
Decreased bed mobility	Weakness and decreased pulmonary and muscular endurance	The patient will gain good bed mobility and endurance within two weeks of intervention	Rolling facilitation and transition training (supine-to-sit, pelvic bridging, and supine-to-long-sitting)
Decreased out-of-bed transitions	Weakness in girdle muscles and decreased stability	Mr. X will get trained in out-of-bed mobility in three weeks	Transition training, supine-to-sit, and sit-to-stand
Impaired Proprioception	Prolong bed rest	Proprioception will be regained with proper training in three weeks	Proprioceptive training and joint compression
Reduced muscle strength	Weakness due to the disease and hospital stay	Mr. X will regain the reduced muscle strength and be able to perform his ADLs by himself within three weeks	Upper limb strength training with a water bottle (1/2 L initially, then progressed to 1 L). Lower limb strength training with weight cuff (½ kg initially, then progressed to 1 kg). Hip hikers strengthening along with quadriceps strengthening
Impaired sitting balance	Prolong bed rest	The patient will regain the sitting balance within three weeks of rehabilitation	Proprioceptive neuromuscular facilitation can be taught – alternating isometrics and rhythmic stabilization. Perturbations in a safe manner, with a variety of surfaces
Impaired fine motor training	Distal weakness	Mr. X will regain fine motor functions by the end of four weeks	Rubber band exercises, stress ball exercises, handwriting practice
Impaired walking pattern	Prolong hospital stay and cerebellar improvement	Mr. X will be able to walk independently in a good walking pattern after 3-4 weeks of gait training	Side leg raises, ankle dorsiflexion, toe raises, heel raises, seated marching, dynamic quadriceps (Figure 1B), knee-to-chest, single-leg stance, squatting, and gait training
Decreased ADL	Decreased performance of muscles	Mr. X will be able to resume college after 5-6 weeks of intervention	Encourage the use of the extremities for ADL

Follow-up and outcome measures

**Figure 1:**
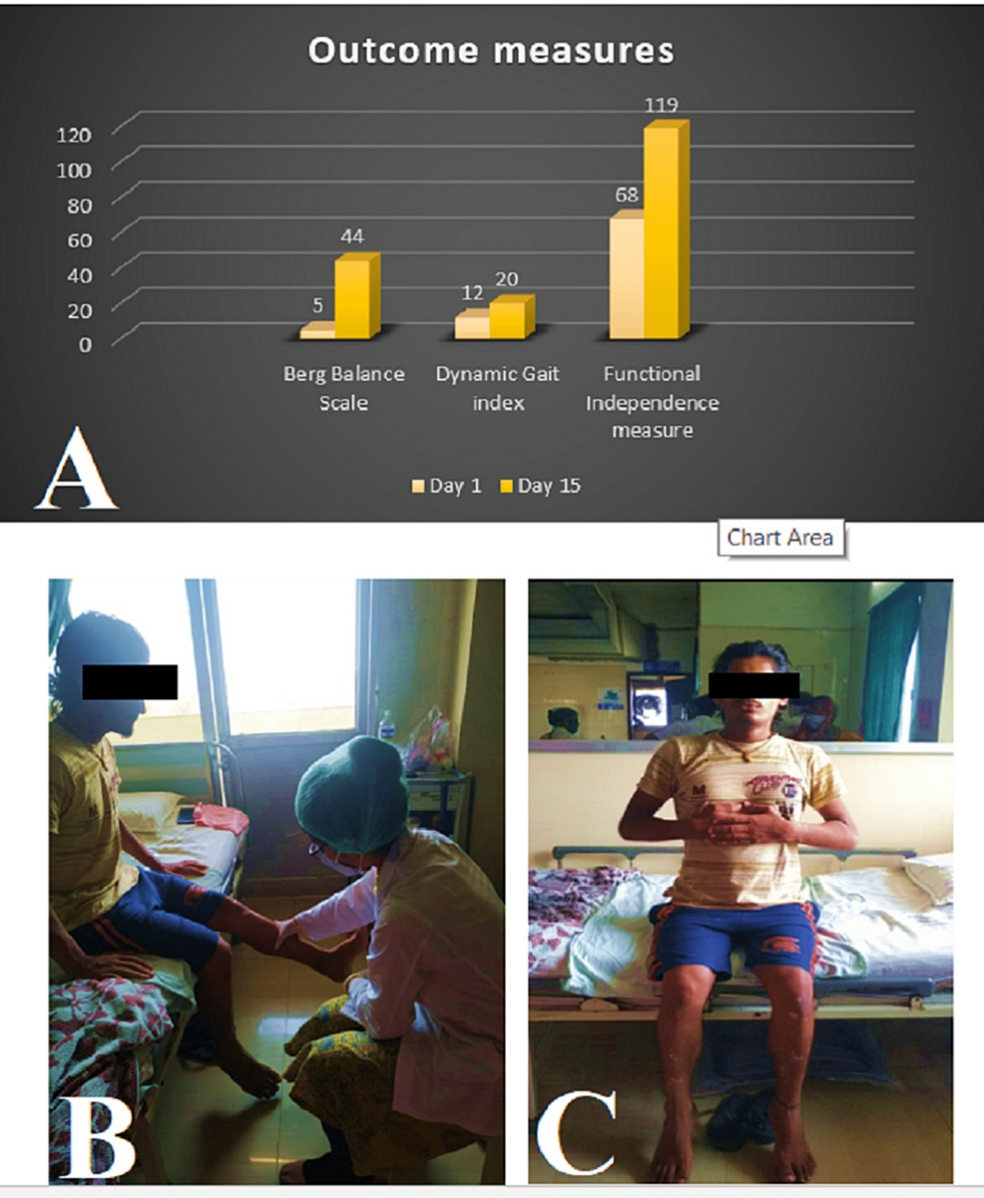
Outcome measures and patient performing exercises. A: Outcome measures on day 1 and day 15 B: Physiotherapy rehabilitation (dynamic quadriceps) C: Patient performing breathing exercise

Incentive spirometry measures increased from 900 cc to 1200 cc over 10 days. There was an improvement in overall hand function, proper grasp, and opposition. The Hughes severity score decreased from 4/6 to 0/6, indicating that normal function was achieved post-rehabilitation. Other outcome measures are given in Figure 1A. Figure 1B shows the patient performing resistance dynamic quadriceps exercises, and Figure 1C shows the patient performing breathing exercises.

Results

The Hughes severity scale normalized to 0/6 (normal) by the time of discharge. The patient was able to perform routine ADL and movement transitions (sit-to-stand, squatting, and stair climbing) that were previously difficult. Dorsiflexion and hip internal rotator power also improved post-rehabilitation. There was an improvement in the Berg balance scale and dynamic gait index which indicate improved static and dynamic balance of the patient. All of this led to the enhancement of the quality of life of the patient and early return back to his occupation.

## Discussion

GBS is defined clinically by the loss of reflexes and an increase in CSF protein content that progresses rapidly. Small action potentials, prolonged distal motor latency, delayed F-waves, and conduction block are all observed neurophysiologically [3]. In this case report, we presented a patient who developed GBS simultaneously with a COVID-19 infection. GBS was confirmed with clinical features, nerve conduction velocity, and CSF analysis, and COVID-19 was confirmed with a nasopharyngeal swab. The first case of COVID-19-associated GBS during the COVID-19 pandemic was identified in Wuhan as a possible para-infectious illness, where the patient had COVID-19 symptoms seven days following the start of GBS symptoms [1]. There have also been numerous case reports in the past indicating a connection between GBS and COVID-19 infections [7,8]. Early diagnosis and rehabilitation are the keys to early recovery. Following a COVID-19 infection, rehabilitation has been shown to enhance patient health outcomes, with fewer complications in the ICU, faster recovery, less disability, easier early discharge, and a lower chance of readmission [9]. With proper medical management and rehabilitation, the path toward early functional independence can be achieved [10].

In this patient, the medical management for COVID-19 was started from the day of diagnosis along with intravenous immunoglobulin for GBS, and physiotherapy was started after the repeat COVID-19 test was negative [11]. Physiotherapy can be started with proper monitoring to prevent respiratory and other complications. Early rehabilitation has been shown to play a positive role in mitigating neurological manifestations of COVID-19 [12]. Cardio-respiratory rehabilitation, early mobilization, frequent posture changes, bed mobility, sit-to-stand exercises, ADL, neuromuscular electrical stimulation, progressive aerobic exercise, and education on energy conservation are all general rehabilitation considerations in the post-acute phase of COVID-19 infection [13]. Other studies have demonstrated an improvement in the Berg balance score and the functional independence of the patient in the setting of comprehensive rehabilitation [14]. The Hughes severity score improved with normalization of the score, indicating the effectiveness of the intervention provided [15].

## Conclusions

Our study concludes that neuro physiotherapy rehabilitation yields positive outcomes and reduces the hospital stay of the patients, making them able to go back to their occupations. The study further demonstrates the importance of the planned physiotherapy protocol in the management of acute cases of GBS along with COVID-19. Amid the COVID-19 pandemic, patients exhibiting paresthesia, tingling feelings, and trouble walking should not be dismissed as simply viral-associated myalgia and arthralgia. Due to COVID-19, GBS should be taken into account as a possible uncommon but serious consequence.
